# A high burden of diabetes and ankle brachial index abnormalities exists in Mexican Americans in South Texas

**DOI:** 10.1016/j.pmedr.2024.102604

**Published:** 2024-01-09

**Authors:** Anand Prasad, Audrey C. Choh, Nelson D. Gonzalez, Marlene Garcia, Miryoung Lee, Gordon Watt, Liliana Maria Vasquez, Susan Laing, Shenghui Wu, Joseph B. McCormick, Susan Fisher-Hoch

**Affiliations:** a The University of Texas Health Science Center San Antonio, San Antonio, TX, USA; b University of Texas School of Public Health Brownsville Regional Campus, USA; c Memorial Sloan Kettering Cancer Center, Department of Epidemiology and Biostatistics, India; d The University of Texas Health Science Center at Houston, USA

**Keywords:** Peripheral arterial disease, Ankle brachial index, Diabetes mellitus, Population health, Amputation, Vascular medicine, Doppler ultrasound

## Abstract

Ethnic differences exist in the United States in the interrelated problems of diabetes (DM), peripheral arterial disease (PAD), and leg amputations. The purpose of this study was to determine the prevalence and risk factor associations for subclinical PAD in a population sample of Mexican Americans using the ankle brachial (ABI) index.

The ABI-High (higher of the two ankle pressures/highest brachial pressure) and ABI-Low (lower of the two ankle pressures/highest brachial pressure) were calculated to define PAD. Toe brachial index (TBI) was also calculated. 746 participants were included with an age of 53.4 ± 0.9 years, 28.3 % had diabetes mellitus (DM), 12.6 % were smokers, and 51.2 % had hypertension (HTN). Using ABI-High ≤ 0.9, the prevalence of PAD was 2.7 %. This rose to 12.7 % when an ABI-Low ≤ 0.9 was used; 4.0 % of the population had an ABI-High > 1.4. The prevalence of TBI < 0.7 was 3.9 %. DM was a significant risk factor for ABI-High ≤ 0.9 and ABI-High > 1.4, and TBI < 0.7. Increased age, HTN, smoking was associated with ABI-High ≤ 0.9, while being male was associated with ABI-High > 1.4. Increased age, smoking, and lower education were all associated with abnormal TBI.

Despite relatively younger mean age than other studied Hispanic cohorts, the present population has a high burden of ABI abnormalities. DM was a consistent risk factor for PAD. These abnormalities indicate an important underlying substrate of vascular and metabolic disease that may predispose this population to the development of symptomatic PAD and incident amputations.

## 1 Introduction

Lower extremity peripheral arterial disease (PAD) is present in over 8 million individuals in the United States – with a prevalence that is expected to rise over the coming decades (Aboyans et al., 2012). The confluence of aging and comorbidities such as diabetes mellitus (DM) and hypertension are key contributors to this increase in PAD. The prevalence and risk factor associations for PAD have largely been derived from white-non-Hispanic populations. However, the second fastest growing demographic within the United States is self-identified as Hispanic and is largely of Mexican American origin (Aguayo-Mazzucato et al., 2019). The state of Texas has the second largest population of Hispanic individuals in the country (Allison et al., 2004). Within Texas, the Lower Rio Grande Valley region of South Texas has a high concentration of Mexican Americans. This region is notable for a higher frequency of major non-traumatic amputations as compared with the national average (Allison et al., 2006, Allison et al., 2009). This amputation rate exists in the context of a high penetrance of obesity and DM (Allison et al., 2009, Allison et al., 2015, Allison et al., 2010). PAD presenting as critical limb ischemia (CLI) is a major contributor to amputations but remains poorly studied not only in the South Texas but also in the Hispanic demographic as a whole.

The purpose of the present study was to determine the prevalence and risk factor associations for subclinical lower extremity PAD using the ankle brachial (ABI) index in a prospectively obtained population sample of Mexican Americans living in South Texas. The Doppler derived ABI was chosen due to existing literature on the utility of this tool to screen populations for prevalent PAD, ease of measurement, and established correlation with atherosclerotic cardiovascular risk factors. The receiver operating characteristics of the ABI measured by Doppler techniques in patients with suspected PAD, cardiac patients and in populations enriched with DM have shown areas under the curve ranging between 0.87 and 0.95 (American, 2020). Given the potential limitations of PAD detection using the ABI in calcified vessels, we also incorporated measurement of the toe brachial index (TBI) (Chen et al., 2022). We examined the relationships between clinical and socioeconomic risk factors and the presence of PAD. Given high rates of DM and obesity in this population, we hypothesized that there would be a high burden of subclinical PAD and that metabolic health would be associated with lower extremity atherosclerosis.

## 2 Methods

### 2.1 Cohort description

Prospective data were collected as part of the Cameron County Hispanic Cohort (CCHC) Study. The details of this population-based study have been published elsewhere (Allison et al., 2010). Briefly, households were selected from randomly selected blocks according to the year 2000 census and adults in randomly selected households were recruited. The CCHC is a community-dwelling, largely Mexican American cohort living in Brownsville (Cameron County), Texas, a city on the lower Rio Grande River at the US-Mexico border. Family, socioeconomic, educational, and personal medical histories were obtained using interview-based questionnaires. Symptoms of claudication were assessed using the San Diego Claudication Questionnaire (Criqui et al., 1996). Surveys and data collection were conducted in the participants’ language of preference (Spanish or English) by bilingual research nurses and field workers. The study was approved by the Committee for the Protection of Human Subjects of the University of Texas Health Science Center of Houston (UT Health) and all subjects provided informed consent.

### 2.2 Study sample

The study sample is a cross-sectional cohort of participants who had bilateral ABI measures at their last visit consisting of 746 participants (248 males, 498 females) who visited the UT Health School of Public Health Brownsville campus, Clinical Research Unit between August 2015 and February 2020 and ranged in age between 18 and 86 years.

### 2.3 Ankle brachial and toe brachial indices

The ABI measurement was first implemented in August 2015 in the CCHC and performed by a skilled technician using a SmartDop 30 EX Doppler system (Koven, St. Louis, MO) using a validated research protocol (Criqui et al., 2010). Subjects were placed in a resting supine position for at least 5 min. An initial non-ABI related cuff brachial blood pressure was performed to acclimatize the subject to the procedure. Cuffs were then applied to the bilateral ankle and brachial positions. In a serial fashion, both brachial pressures, followed by bilateral dorsalis pedis (DP) and posterior tibial (PT) pressures and waveforms were obtained. The higher of the two brachial pressures was used for the present analysis in all index calculations. For a given limb, ABI-High was calculated as the higher of the two pedal pressures divided by the highest brachial pressure and ABI-Low was calculated as the lower of the two pedal pressures divided by the highest brachial pressure. We chose to examine both the ABI-High and the ABI-Low as prior studies have demonstrated that the magnitude of risk factor associations, relationship to ethnicity and sensitivity for PAD in population studies varies by definition used (11). TBI was measured with the same system using an integrated photoplethysmography probe. ABI or TBI for a given participant was considered normal only if both legs were normal. Normal was considered to be any ABI > 0.9 and ≤ 1.4 or TBI ≥ 0.7 (1). Abnormal ABI values include poorly compressible vessels - defined as an ABI-High > 1.4 or PAD – defined as ABI values ≤ 0.9. Critical values were used to create dependent variables; 1) ABI-Low ≤ 0.9, 2) ABI-High ≤ 0.9, 3) ABI-High > 1.4, 4) abnormal TBI (TBI < 0.7), and 5) any abnormal ABI, according to the American Heart Association/American College of Cardiology Guidelines (1).

### 2.4 Risk factor definitions

Methodology to assess clinical history, risk factors, and exam parameters have been outlined previously (7). Socio-demographic variables included annual household income, education, health insurance, and marital status. Cardiovascular disease (CVD) was defined as having had a heart attack, having had a carotid endarterectomy, coronary bypass surgery, or a coronary stent procedure. DM was defined as either self-reported DM, use of diabetic medications, a measurement of glycated hemoglobin (HbA1c) > 6.5 %, or mean fasting blood glucose (FBG) ≥ 126 mg/dL (Criqui et al., 2005). Pre-diabetes (Pre-DM) was defined as 100 ≤ FBG < 126 mg/dL or 5.7 % ≤ HbA1c < 6.5 % (12). Hypertension (HTN) was defined as use of anti-hypertensive medication or if the systolic blood pressure (SBP) was ≥ 130 mmHg or the diastolic blood pressure (DBP) was ≥ 80 mmHg (Cronenwett and Birkmeyer, 2000). Chronic kidney disease (CKD) was defined as stage 3 or greater using an estimated glomerular filtration rate (eGFR) < 60 mL/min/1.73 m^2^ by the Modification of Diet in Renal Disease (MDRD) equation (De Vita CJ, Pollard KM. Increasing diversity of the U.S. population. Stat Bull Metrop Insur Co., 1996). Prior histories of myocardial infarction (MI), foot ulcer, stroke or transient ischemic attack were self-reported. A smoker was defined as the use of at least 100 cigarettes in a lifetime and reporting being a current smoker. Body mass index (BMI, kg/m^2^) was calculated as weight in kilograms divided by height in meters. Overweight was defined as having body mass index (BMI) between 25 to 29.0 kg/m^2^, while obesity was defined having BMI ≥ 30.0 kg/m^2^ (Fisher-Hoch et al., 2010). Waist to Hip Ratio (WHR) was calculated as waist circumference divided by hip circumference. Dyslipidemia defined as HDL ≤ 40 or 50 mg/dL (males or females respectively), LDL ≥ 130 mg/dL, total cholesterol (TC) ≥ 200 mg/dL, or triglyceride (TG) levels ≥ 150 mg/dL.

Participants not meeting criteria for HTN or DM were categorized as normal. Participants who met diagnostic criteria for DM or HTN but were not on medications were considered “untreated”. In contrast, participants who were taking medications but had BP < 130/80 mmHg, and HbA1c < 6.5 % and FBG < 126 mg/dL were considered to be “controlled”, while participants who still exceeded diagnostic criteria for HTN and DM despite taking medication were considered to be “uncontrolled”.

### 2.5 Statistical analysis

The analyses took into consideration clustering effects of multiple participants from the same household as well as census block. This adjustment for clustering effects has previously been defined in detail (Garcia et al., 2019). Data are summarized as mean and standard error for continuous variables and frequency and percentages for categorical variables using PROC SURVEYEMEANS. Univariable associations between the four ABI variables and all demographic, biologic, social and clinical variables using PROC SURVEYLOGISTIC. PROC SURVEYFREQ was used to examine the relationship between PAD traits and polychotomous variables using Rao-Scott chi-square test. Multivariable-adjusted survey-weighted binomial logistic regression analyses were performed to obtain the weighted odds ratios (OR) and 95 % confidence intervals (CIs) for ABI values (versus normal values) and predictor variables. Predictor variables that were highly skewed were transformed to nearly normal for model stability. We initially started with a multivariable-adjusted model that included age, sex, HTN, DM, smoking, CKD, WHR, log (HDL), inverse log(TG), and LDL to explore the relationships between predictor variables and the four PAD traits. HDL and TG were highly skewed and transformed for model stability. Because there were no smokers who had ABI-High > 1.4, smoking was eliminated in all ABI-High > 1.4 models. Variables were entered and removed sequentially to arrive at the final model. The analysis model included biological variables as well as demographic and social risk factors. All statistical tests were two-sided and performed using SAS 9.4 (SAS Institute Inc., Cary, NC).

## 3 Results

### 3.1 Subject characteristics

A total of 746 participants were enrolled. The weighted demographic and clinical variables are shown in Table 1. Of the study population, 98.1 % were self-identified as Mexican American. The mean age was 53.4 ± 0.9 years and 66.8 % ± 2.3 % of the study population were female. The prevalence of cardiovascular risk factors was as follows: DM (28.3 %), pre-DM (40.7 %), HTN (51.2 %), obesity (50.0 %), and overweight (35.2 %). The prevalence of CKD, CVD, stroke or TIA, and foot amputation or ulcer was 3.6 %, 3.6 %, 3.2 %, and 2.1 % respectively. Only 1 subject reported typical claudication based on the San Diego Claudication Questionnaire (Gerhard-Herman et al., 2017). The prevalence of uncontrolled DM and HTN was 15.6 % and 14.4 %, respectively. The prevalence of newly diagnosed DM and HTN were 9.7 % and 19.6 %, respectively.

**Table 1:** Weighted subject characteristics evaluated for peripheral arterial disease by the ankle brachial index in the Cameron County Hispanic Cohort, 2015–2020.

	Sample (N)	Mean ± SE	Range
**Continuous variables (Mean ± SE)**			
Age (years)	746	53.4 ± 0.9	18.0–86.0
Sex (female)^**^	746	66.8 % ± 2.3	–
Body mass index (BMI, kg/m^2^)	746	30.8 ± 0.4	16.2–68.9
Waist circumference (cm)	746	103.2 ± 0.8	37.4–178.0
Waist Hip Ratio (WHR)	746	0.95 ± 0.00	0.40–1.48
Fasting blood glucose (mg/dL)	737	108.7 ± 1.7	66.0–374.0
Glycated Hemoglobin (HbA1c, %)	741	6.3 ± 0.1	4.5–13.1
Insulin	633	12.4 ± 0.6	1.3–73.0
Homeostatic Model Assessment for Insulin Resistance (HOMA-IR)	628	3.4 ± 0.2	0.02–32.2
High density lipoprotein cholesterol (HDL, mg/dL)	736	48.2 ± 0.7	23.0–109.0
Low density lipoprotein cholesterol (LDL, mg/dL)	729	105.1 ± 1.5	13.0–231.0
Total cholesterol (TC, mg/dL)	736	182.4 ± 1.8	50.0–303.0
Triglycerides (TG, mg/dL)	740	147.7 ± 4.6	33.0–1,596.0
Systolic blood pressure (SBP, mmHg)	744	121.1 ± 0.9	83.0–200.0
Diastolic blood pressure (DBP, mmHg)	744	72.2 ± 0.4	47.0–108.0
Carotid intimal thickness (cIMT, mm)	431	0.72 ± 0.01	0.43–1.35
MDRD glomerular filtration rate (mL/min)	735	100.3 ± 1.3	6.0–205.0
Education (years)	746	10.7 ± 0.2	0.0–20.0
Income ($ x 1,000)	623	30.26 ± 3.99	0.01–2,600
**Categorical variables (%, SE)**			
Dyslipidemia	738	72.1 ± 2.3	–
Non-LDL dyslipidemia (high TG or low HDL)	740	58.5 ± 2.6	–
Hypertension	744	51.2 ± 2.6	–
Diabetes	744	28.3 ± 2.4	–
Pre-diabetes	744	40.7 ± 2.6	–
Overweight	746	35.2 ± 2.4	–
Obese	746	50.0 ± 2.6	–
Stroke or transient ischemic attack	746	3.2 ± 0.8	–
Cardiovascular disease	746	3.6 % ± 0.9	–
Chronic kidney disease (CKD, eGFR ≤ 60 mL/min)	746	3.6 % ± 1.0	–
Smoker (currently smokes or 100 cigarettes/lifetime)	746	12.6 % ± 1.7	–
Formerly married (vs married, single)	744	20.6 % ± 2.4	–
History of foot ulcer or toe/limb amputation (yes)	723	2.1 % ± 0.9	–
**PAD measurements (%, SE)**			
ABI-Low ≤ 0.9	746	12.7 ± 1.6	–
ABI-High ≤ 0.9	746	2.7 ± 0.8	–
ABI-High > 1.4	746	4.0 ± 1.0	–
TBI < 0.7	745	3.9 ± 0.9	–
Any evidence of PAD^**^	746	16.7 ± 1.8	–

*Sex is unweighted in order to be consistent with reporting of real numbers of males and females in the study. **Any abnormal ABI or TBI calculation.

### 3.2 PAD prevalence

Using an ABI-High ≤ 0.9, the prevalence of PAD was 2.7 % ± 0.8 % in the sample. 4.0 % ± 1.0 % of the study population had an ABI-High > 1.4. The prevalence of PAD rose to 12.7 % ± 1.6 % when an ABI-Low ≤ 0.9 was used. Approximately 3.9 % ± 0.9 % had an abnormal TBI. The prevalence of any evidence of PAD (any abnormal ABI or TBI measure) was 17.8 % ± 1.9 %.

### 3.3 Associations between cardiovascular risk variables, demographic and social factors with ABI defined PAD

After initial univariable logistic regression modeling, the influence of risk factors on PAD categories were explored in a multivariable model which included cardiovascular risk factors as well as the social variables of education, marital status, having insurance and income (Table 2). After adjusting for social variables, increased age, smoking, and DM was associated with higher odds of having ABI-High ≤ 0.9 and TBI < 0.7. DM or being male was also associated with higher odds of ABI-High > 1.4. Additional exploratory analyses for gender and ABI > 1.4 are shown in the Supplementary Tables 1 and 2. ABI > 1.4 was significantly more prevalent in males vs females (7.7 % ± 2.3 vs 1.5 % ± 0.5, P < 0.0001). After multivariable adjustment, female gender was negatively associated with an ABI > 1.4 (P < 0.01). Among the social variables, not having a high school degree was associated with increased odds of ABI-High ≤ 0.9 and TBI < 0.7, while lower income was associated with greater odds of having ABI-High > 1.4. Marital status was not associated with any PAD variables. In multivariable analysis, an ABI-Low ≤ 0.9 did not demonstrate association with any additional variables.

**Table 2:** Relationship of biological, demographic and social risk factors on measurements of peripheral arterial disease evaluated by the ankle brachial and toe brachial indices in the Cameron County Hispanic Cohort, 2015–2020.

	ABI-Low ≤ 0.9 vs. Normal*	ABI-High ≤ 0.9 vs. Normal*	ABI-High > 1.4 vs. Normal*	TBI < 0.7 vs. Normal*	Any ABI/TBI vs Normal*
	**Estimated Odds Ratios (95 %CI)**				
Age	1.02 (0.99,1.05)	1.17 (1.07,1.28)	0.98 (0.94,1.02)	1.15 (1.08,1.23)	1.01 (0.98,1.04)
Sex (females)	1.19 (0.60,2.36)	0.57 (0.11,3.01)	0.14 (0.05,0.43)	0.91 (0.22,3.73)	0.68 (0.37,1.22)
Hypertension	1.22 (0.66,2.26)	6.06 (1.27,28.83)	0.54 (0.16,1.79)	1.79 (0.56,5.68)	1.02 (0.55,1.88)
Smoker	2.21 (0.93,5.23)	5.56 (1.23,25.24)	–	6.94 (1.72,27.97)	1.50 (0.65,3.43)
Diabetes	1.10 (0.60,2.04)	3.58 (1.14,11.28)	3.45 (1.19,10.00)	3.72 (1.40,9.87)	1.43 (0.83,2.45)
≤ High school degree	0.55 (0.30,1.04)	0.06 (0.02,0.26)	1.24 (0.37,4.14)	0.25 (0.08,0.83)	0.70 (0.39,1.27)
Formerly married (vs single/married	1.66 (0.75,3.64)	1.80 (0.34,9.44)	0.66 (0.16,2.69)	2.30 (0.62,8.61)	1.49 (0.72,3.08)
Has insurance	0.88 (0.42,1.84)	3.50 (0.84,14.57)	3.06 (0.93,10.12)	2.96 (0.67,13.00)	1.22 (0.57,2.62)
Income (in $1000)	1.00 (0.98,1.01)	0.97 (0.94,1.01)	0.93 (0.89,0.97)	0.98 (0.94,1.02)	0.98 (0.96,1.00)

*Normal defined as 0.9 < ABI ≤ 1.4 or TBI ≥ 0.7.

## 4 Discussion

In this population sample of Mexican Americans, we demonstrate a high prevalence of pre-clinical PAD with 16.7 % of the sample having some abnormality in the ABI or TBI. DM, smoking and increased age were significant risk factors for the presence of PAD. Hispanics of Mexican origin represent a growing population within the United States. This group is prone to the development of obesity and DM (Goodney et al., 2013). The causative factors for these findings are still being elucidated and may include potential genetic variants as well as socioeconomic challenges with respect to access to medical care (Goodney et al., 2013). While the underlying substrate of DM may have heritable factors – the influence of genetics on PAD risk remain poorly defined (Hendriks et al., 2016). The following discussion will place the present results into the context of the published literature.

### 4.1 Prior studies of subclinical PAD in Hispanics

These data reveal the extent of underlying lower limb pathology in a population with one of the highest rates of amputations in the United States (Allison et al., 2006, Allison et al., 2015, Herraiz-Adillo et al., 2020). For reference, Medicare derived data on major amputations in the Rio Grande Valley demonstrate amputation rates in diabetics of over 1.5 times the national average (Herraiz-Adillo et al., 2020). The presence of a high burden of subclinical disease and DM may be important substrates for incident amputations in this region. Despite younger mean age, the present cohort has comparable or higher prevalence of PAD as compared to prior studies of PAD in Hispanic populations. These studies are summarized in Table 3. The Hispanic Community Health Study - Study of Latinos (HCHS-SOL) reported a prevalence of PAD was 3.2 % in the Mexican American subgroup (ABI-High < 0.9) and 2.6 % for an ABI-High ≥ 1.4 (Hoyer et al., 2013). Significant independent risk factors for PAD included age, female gender, hypertension, DM, coronary heart disease, sleep apnea, and a history of smoking. The Multi-Ethnic Study of Atherosclerosis (MESA) reported a prevalence of 2.4 % (ABI-High < 0.9) in Hispanics (Kellogg et al., 2019). In the primary PAD analysis in MESA, individuals with established cardiovascular disease (CVD) were excluded, as were individuals with ABIs > 1.4. The San Diego Population Survey Study assessed the presence of clinical and subclinical PAD in a retired University Population and reported a prevalence of PAD in Hispanics of 1.8 % (6 subjects) (23). The study did not include ABIs > 1.4 and was composed of highly selected, older insured, current or retired University of California San Diego employees. The National Health and Nutrition Examination Survey (NHANES) found a prevalence of PAD (ABI-High < 0.9) of 3 % in the Hispanic group (Levey et al., 1999). The study excluded individuals with elevated ABIs, and Hispanics made up the smallest ethnic group in the analysis (9.1 %). In summary, these studies had variable numbers of Mexican Americans and individuals with DM, and they largely limited the definition of PAD to ABI-High < 0.9.

**Table 3:** Summary of published studies on peripheral arterial disease prevalence in population cohorts with an emphasis on the Hispanic subgroups.

Study	Cohort Sample size	Demographic Details	Prevalence and Definitions of PAD
Hispanic Community Health Study – Study of Latinos (HCHS-SOL)	n = 9,648	• Mean age 56 years • 34 % Mexican American • 27 % with diabetes	• Mexican American Subgroup: • 3.2 % using ABI-High < 0.9 2.6 % using ABI-High ≥ 1.4
The Multi-Ethnic Study of Atherosclerosis (MESA)	n = 6,814	• Mean age 62 years • 14 % with diabetes	• Hispanic Subgroup: 2.4 % using ABI-High < 0.9
San Diego Population Study	n = 2,343	• Mean age 60 years • 6.9 % with diabetes	• Hispanic Subgroup: 1.8 % using ABI-High < 0.9
National Health and Nutrition Examination Survey (NHANES)	n = 3,348	• All subjects over age 40 years • 10.8 % with diabetes	• Hispanic Subgroup: 3.0 % using ABI-High < 0.9

Our study has several key differences – providing further insight into the prevalence of preclinical disease in Mexican Americans. The CCHC population is self-identified as Mexican American and therefore culturally and ethnically less heterogeneous than other studies which included multiple Hispanic subgroups (Hoyer et al., 2013, Laing et al., 2015, Levey et al., 1999, Lu et al., 2014). The CCHC also uses random population sampling (as opposed to advertised enrollment widely used in most prior studies) and therefore represents a true community-dwelling Mexican American cohort. Lastly, it should be emphasized that the CCHC represents a group of subjects with poor access to medical care, low educational level, and endemic poverty – sharply contrasting with the Hispanic cohorts noted above.

### 4.2 Calculation methods of the ABI: Impact on PAD prevalence and risk factors

In our study, we found a substantially higher prevalence of PAD using the ABI-Low in comparison to the ABI-High. The ABI-High has traditionally been used in clinical settings for the diagnosis of PAD. However, since the ABI-High uses the higher of the two pedal pressures, disease or occlusion of a tibial vessel may be missed. This limitation of the ABI-High may potentially be magnified in populations that have an increased frequency of infrapopliteal disease. Conversely, the ABI-Low may overestimate disease presence when there is measurement error (inability to locate an artery) or congenital absence of a tibial vessel. The impact of using these calculations on the prevalence and risk factor associations was examined by Allison et al. using data from the MESA population (Criqui et al., 2010). In their Hispanic cohort, they demonstrated a prevalence of PAD by ABI-High < 0.9 of 2.1 % - rising to 5.9 % when using ABI-Low. In the overall population, the relative increase in PAD prevalence was 3–4 times higher across genders when using the ABI-Low. When taken in context of other measures of subclinical plaque (coronary calcium or carotid plaque), the ABI-Low had the greatest sensitivity and predictive accuracy for atherosclerosis. In contrast, the ABI-High had greater specificity, and stronger associations with CVD risk factors. Consistent with these data, our findings demonstrated increased prevalence of disease but loss of the risk factor associations of age, DM and smoking when using ABI-Low as compared to the ABI-High.

### 4.3 Risk factors for PAD

In the present study, both aging and DM remained significant risk factors for an ABI-High, regardless of the direction. Smoking was strongly associated with an ABI-High ≤ 0.9. Age, DM, and smoking are established ‘traditional’ risk factors of lower extremity PAD in multiples studies – largely derived from white populations (Newhall et al., 2016). There are limited data on clinical risk factors associated with PAD in Mexican Americans in the literature. Comparative studies amongst different Latino groups suggest that when adjusted for established risk factors for PAD, Hispanic ethnic subtype may be important for the presence of subclinical PAD. For example, when compared to Mexican Americans, Cuban Americans appear to have higher odds ratio for the presence of PAD (Hoyer et al., 2013). These data would imply that beyond traditional risk factors, genetic, environmental, and unmeasured influences might affect PAD development in Latinos. The question remains as to whether additional novel risk factors might be relevant to the presence of PAD in this population. Specifically, exploration of potential genetic markers related to PAD remains a future research interest in this cohort (Hendriks et al., 2016).

### 4.4 Factors associated with an ABI > 1.4 (poorly compressible arteries)

We noted the prevalence of an elevated ABI (ABI-High > 1.4) of 4.0 % - with the primary risk factors being male gender, DM and income. This is substantially higher than that seen in Mexican Americans in the HCHS-SOL as described earlier (2.6 %) (Hoyer et al., 2013). This examination of a diverse group of Latino subjects, noted that odds of an elevated ABI did not vary by Hispanic subgroup, and male gender was also a consistent risk factor for elevated ABIs. The variable presence of poorly compressible vessels has been shown in multiple populations. In a population study (n = 4,393, mean age 58 years) of Native Americans (Strong Heart Study) – highly enriched with DM (∼40 %), the prevalence of ABI-High > 1.4 was 9.2 % (Resnick et al., 2004). A sample of patients presenting for routine exams in primary care clinics in Italy (n = 9647), demonstrated a prevalence of elevated ABI-HI of 7.6 %, associated with age, male gender, HTN and DM (28). In a group of individuals (n = 7542) with established CVD or at high risk of CVD (SMART study), the prevalence of elevated ABI was 4.5 %, with multiple significant risk factors including age, male gender, DM, increased BMI, and elevated non-HDL cholesterol (Selvin and Erlinger, 2004). In a Japanese cohort of patients with established CVD (n = 2,419) and penetrance of DM of 25 %, an elevated ABI was found in only 2.6 % of subjects (Signorelli et al., 2011). Age, gender, and DM were not associated with an elevated ABI, while hemodialysis was the only significant risk factor.

Although our sample size of males in the present study was small, there was a notable gender difference in the ABI > 1.4 measurement. Male gender was associated with an ABI > 1.4 which was persistent on multivariable analyses. Males did have a higher prevalence of pre-existing CVD and CKD in the cohort – both factors associated with vascular calcium. No other ABI variables were different by gender. In the MESA cohort, males were more likely to have an ABI > 1.4 (Stepler and Lopez, 2016). In that study, an elevated ABI also predicted CVD events. These findings suggested a hypothesis that the elevated ABI may be a marker for arterial stiffness and possibly subclinical atherosclerosis which portends clinical events. These data are also consistent with the free-living population study of largely middle aged adults by Chen et al which also demonstrated a much higher rate of vascular calcification in males versus females (Wang et al., 2005). Additionally, a cross-sectional analysis of X-ray detected vascular calcification in 650 asymptomatic primary care patients demonstrated a gender and age-related prevalence of calcium. Calcium was more common in men as compared to women across age quartiles (<50, 50–60, 60–70, and > 70 years). However, the prevalence markedly rose in women after menopause (>50 years) (Watt et al., 2016).

Taken together, the published data suggest that the frequency and risk factors for an elevated ABI varies considerably according to the study cohorts (population sample versus general patients versus CVD patients) and by age and gender. DM appears to be a common but not universal risk factor for ABI > 1.4 in the literature. There are several potential explanations for the lack of association of DM and ABI > 1.4 in the present study. The CCHC subject cohort is relatively young, with a lower prevalence of DM than for example in the Strong Heart Study (Resnick et al., 2004). An increased ABI may be related to medial artery calcification. It is postulated that this process is aging dependent and worsens over time. In addition, there may be important ethnic differences in elevated ABI with respect to prevalence, risk factor associations, and progression of vessel stiffening with aging (Lu et al., 2014). The precise mechanisms for the sex-based differences with respect to vascular stiffening; medial calcification and elevation of the ABI remain to be elucidated. In conditions of an elevated ABI > 1.4, the use of the TBI may be helpful to examine for underlying PAD (Chen et al., 2022). In the present study, DM and smoking were the most consistent risk factors for an abnormal TBI. The diagnostic threshold for an abnormal TBI remains poorly defined. A value of < 0.70 as cutoff has been used in a variety of studies in the literature without rigorous validation (Whelton et al., 2018). The distribution of abnormal TBIs in an unselected population-based cohort has not been well defined to date. The role of TBIs for PAD in this context remains uncertain.

## 5 Study limitations

The present cross-sectional analysis was derived from a population of exclusively Mexican Americans in Cameron County, Texas. Therefore, comparisons to other ethnic groups in the region were not possible. In addition, applicability of the findings to other Mexican American clusters or Latinos may or not be valid. The study used data on self-reported DM, diabetic medications or an abnormal fasting glucose or HbA1C% rather than more sensitive metabolic testing may have underestimated the true prevalence of DM. The sample size was also modest with infrequent history of prior amputations or intermittent claudication symptoms because we recruited ambulatory participants who are able to visit the study clinic without assistance. Therefore, further studies are required to confirm our findings and to understand the associations with incident CLI. Lastly, the sample size of males is too small to derive meaningful analyses based on gender analyses.

## 6 Conclusions

The prevalence of abnormalities of the ABI in this relatively young Mexican American cohort was higher than that reported in several prior studies in Hispanic populations. These abnormalities included both sensitive and specific measures of PAD as well as elevations in the ABI associated with vessel stiffening. These data support an underlying substrate that may predispose this population to the development of symptomatic PAD. The causality of our ABI findings with incident limb symptoms and amputations remains to be studied and will be prospectively explored in this population cohort.

## Author contributions

Anand Prasad (study design, data analysis, manuscript drafting and editing).

Audrey C. Choh (data analysis, manuscript editing).

Nelson D. Gonzalez (data analysis, data collection, manuscript editing).

Marlene Garcia (manuscript editing, study design).

Miryoung Lee (data analysis, manuscript editing).

Gordon Watt (data collection, data analysis, manuscript editing).

Liliana Maria Vasquez (data collection, data analysis, manuscript editing).

Susan Laing (manuscript editing, study design).

Shenghui Wu (data analysis, manuscript editing).

Joseph B. McCormick (manuscript editing, study design).

Susan Fisher-Hoch (manuscript editing, study design).

## Funding

This work was supported by the Mike Hogg Fund, Freeman Heart Association, and Center for Clinical and Translational Sciences, which is funded in part by National Institutes of Health Clinical and Translational Award UL1 TR000371 from the National Center for Advancing Translational Sciences. The content is solely the responsibility of the authors and does not necessarily represent the official views of the National Center for Advancing Translational Sciences or the National Institutes of Health. There are no conflicts of interest for any of the authors with commercial interests.

## CRediT authorship contribution statement

**Anand Prasad:** . **Audrey C. Choh:** Writing – review & editing, Writing – original draft, Validation, Supervision, Formal analysis, Data curation. **Nelson D. Gonzalez:** Formal analysis, Data curation. **Marlene Garcia:** Writing – original draft, Methodology, Investigation, Formal analysis. **Miryoung Lee:** Writing – review & editing, Writing – original draft, Validation, Methodology, Data curation. **Gordon Watt:** Writing – original draft, Investigation, Formal analysis, Data curation. **Liliana Maria Vasquez:** . **Susan Laing:** Writing – review & editing, Writing – original draft, Resources, Project administration, Methodology, Investigation, Funding acquisition, Data curation. **Shenghui Wu:** Writing – original draft, Validation, Formal analysis. **Joseph B. McCormick:** Writing – original draft, Resources, Project administration, Investigation, Conceptualization. **Susan Fisher-Hoch:** Writing – review & editing, Writing – original draft, Resources, Project administration, Methodology, Investigation, Funding acquisition, Data curation.

## Declaration of competing interest

The authors declare that they have no known competing financial interests or personal relationships that could have appeared to influence the work reported in this paper.

## Data Availability

The data that has been used is confidential.
