# Supplementary material for: A high burden of diabetes and ankle brachial index abnormalities exists in Mexican Americans in South Texas
Source: Prev Med Rep. 2024 Jan 9;38:102604. doi: 10.1016/j.pmedr.2024.102604 (PMC10874877; doi:10.1016/j.pmedr.2024.102604)
Supplement: Supplementary data 1 [file mmc1.docx]

**Supplementary Table 1. Gender differences in baseline characteristics**

|  | Males (n=248) |  | Females (n=498) |  |  |
| --- | --- | --- | --- | --- | --- |
|  | Mean  ± SE | Range | Mean  ± SE | Range | Sex  diff |
| **Continuous variables (Mean ± SE)** |  |  |  |  |  |
| Age (years) | 52.2 ± 1.3 | 18.0 - 85.0 | 54.3 ± 0.9 | 18.0 - 86.0 | 0.1387 |
| Body mass index (BMI, kg/m^2^) | 30.9 ± 0.5 | 18.5 - 48.1 | 30.8 ± 0.4 | 16.2 - 68.9 | 0.9071 |
| Waist circumference (cm) | 105.3 ± 1.1 | 70.6 - 145.6 | 101.7 ± 1.1 | 37.4 - 178.0 | 0.0182 |
| Waist Hip Ratio (WHR) | 0.97 ± 0.01 | 0.77 - 1.19 | 0.93 ± 0.00 | 0.40 - 1.48 | <.0001 |
| Fasting blood glucose (mg/dL) | 110.6 ± 2.9 | 66.0 - 354.0 | 107.3 ± 2.0 | 72.0 - 374.0 | 0.354 |
| Glycated Hemoglobin (HbA1c, %) | 6.3 ± 0.1 | 4.5 - 12.9 | 6.3 ± 0.1 | 4.7 - 13.1 | 0.8621 |
| Insulin | 12.9 ± 1.1 | 1.3 - 59.1 | 12.0 ± 0.5 | 1.3 - 73.0 | 0.4569 |
| Homeostatic Model Assessment for Insulin Resistance (HOMA-IR) | 3.5 ± 0.3 | 0.1 - 21.6 | 3.3 ± 0.2 | 0.0 - 32.2 | 0.485 |
| High density lipoprotein cholesterol (HDL, mg/dL) | 43.4 ± 0.7 | 23.0 - 94.0 | 51.5 ± 1.0 | 26.0 - 109.0 | <.0001 |
| Low density lipoprotein cholesterol (LDL, mg/dL) | 105.3 ± 2.6 | 17.3 - 231.0 | 104.9 ± 1.9 | 13.0 - 204.0 | 0.9193 |
| Total cholesterol (TC, mg/dL) | 181.6 ± 3.0 | 50.0 - 303.0 | 183.0 ± 2.2 | 97.0 - 303.0 | 0.6944 |
| Triglycerides (TG, mg/dL) | 163.5 ± 8.5 | 33.0 - 1,429.0 | 136.9 ± 4.8 | 36.0 - 1,596.0 | 0.0067 |
| Systolic blood pressure (SBP, mmHg) | 122.9 ± 1.5 | 88.0 - 185.0 | 119.9 ± 1.1 | 83.0 - 200.0 | 0.0928 |
| Diastolic blood pressure (DBP, mmHg) | 74.4 ± 0.7 | 51.0 - 108.0 | 70.7 ± 0.5 | 47.0 - 103.0 | <.0001 |
| Carotid intimal thickness (cIMT, mm) | 0.76 ± 0.02 | 0.47 - 1.35 | 0.70 ± 0.01 | 0.43 - 1.23 | 0.0009 |
| MDRD glomerular filtration rate (mL/min) | 97.2 ± 2.2 | 9.0 - 205.0 | 102.5 ± 1.5 | 6.0 - 187.0 | 0.0365 |
| Education (years) | 11.0 ± 0.3 | 0.0 - 20.0 | 1053.8% ± 27.5 | ~5248 | 0.2152 |
| Income ($ x 1,000) | 40.76 ± 9.58 | 0.01 - 2,600.00 | 23.2 ± 1.5 | 0.4 - 180.0 | 0.0691 |
| **Categorical variables (%, SE)** |  |  |  |  |  |
| Dyslipidemia | 68.7% ± 4.1 |  | 74.5% ± 2.7 |  | 0.2260 |
| Non-LDL dyslipidemia (high TG or low HDL) | 54.0% ± 4.4 |  | 61.5% ± 3.1 |  | 0.1604 |
| Hypertension | 52.7% ± 4.3 |  | 50.2% ± 3.1 |  | 0.6237 |
| Diabetes | 46.6% ± 5.4 |  | 48.3% ± 4.3 |  | 0.8027 |
| Pre-diabetes | 60.2% ± 5.3 |  | 54.2% ± 3.6 |  | 0.3328 |
| Overweight | 41.5% ± 4.3 |  | 30.9% ± 3.0 |  | 0.0471 |
| Obese | 47.9% ± 4.4 |  | 51.5% ± 3.1 |  | 0.5108 |
| Stroke or transient ischemic attack | 4.0% ± 1.6 |  | 2.7% ± 0.8 |  | 0.4389 |
| Cardiovascular disease | 6.4% ± 1.9 |  | 1.7% ± 0.5 |  | 0.0011 |
| Chronic kidney disease (CKD, eGFR ≤ 60 mL/min) | 5.9% ± 2.4 |  | 2.1% ± 0.5 |  | 0.0221 |
| Smoker (currently smokes or 100 cigarettes/lifetime) | 18.7% ± 3.0 |  | 8.4% ± 1.8 |  | 0.0019 |
| Formerly married (vs married, single) | 10.1% ± 2.5 |  | 26.3% ± 3.4 |  | <.0001 |
| History of foot ulcer or toe/limb amputation (yes) | 3.3% ± 2.0 |  | 1.3% ± 0.7 |  | 0.2281 |
| **PAD measurements (%, SE)** |  |  |  |  |  |
| ABI-Low ≤ 0.9 | 10.4% ± 2.4 |  | 14.4% ± 2.0 |  | 0.2167 |
| ABI-High ≤ 0.9 | 4.1% ± 1.7 |  | 1.7% ± 0.6 |  | 0.1038 |
| ABI-High > 1.4 | 7.7% ± 2.3 |  | 1.5% ± 0.5 |  | <.0001 |
| TBI < 0.7 | 4.7% ± 1.7 |  | 3.3% ± 0.9 |  | 0.4266 |
| Any evidence of PAD^*^ | 19.1% ± 3.2 |  | 16.9% ± 2.1 |  | 0.5390 |

Supplementary Table 2. Association of gender with ABI calculations

|  | | | | | |  |
| --- | --- | --- | --- | --- | --- | --- |
|  | **ABI-Low ≤ 0.9**  **vs. Normal§** | **ABI-High ≤ 0.9**  **vs. Normal§** | **ABI-High > 1.4**  **vs. Normal§** | | **TBI < 0.7 vs.**  **Normal§** | **Any ABI / TBI**  **vs Normal§** |
|  | **Parameter estimates ± standard error** | | | | |  |
| Sex (females) | 0.17 ± 0.35 | -0.56 ± 0.85 | -1.94 ± 0.56** | | -0.10 ± 0.72 | -0.39 ± 0.30 |
|  | **Estimated Odds Ratios (95 %CI)** | | | | |  |
| Sex (females) | 1.19 (0.60,2.36) | 0.57 (0.11,3.01) | | 0.14 (0.05,0.43) | 0.91 (0.22,3.73) | 0.68 (0.37,1.22) |
